# Using autologous intrauterine platelet-rich plasma to improve the reproductive outcomes of women with recurrent implantation failure

**DOI:** 10.5935/1518-0557.20190055

**Published:** 2020

**Authors:** Fateme Aghajanzadeh, Sedighe Esmaeilzadeh, Zahra Basirat, Treza Mahouti, Fateme Nadi Heidari, Masoumeh Golsorkhtabaramiri

**Affiliations:** 1 Fatemezahra Infertility and Reproductive Health Research Center, Health Research Institute, Babol University of Medical Science, Iran, Middle East

**Keywords:** platelet-rich plasma, embryo transfer, pregnancy rate

## Abstract

**Objective:**

Reproductive clinics are often faced with cases of repeated implantation failure (RIF). This study evaluated whether platelet-rich plasma (PRP) might improve the implantation outcomes of patients suffering from RIF.

**Methods:**

Thirty women with RIF submitted to frozen-thawed embryo transfers were included in the study. Intrauterine infusions of autologous purified platelet preparations were administered 48 hours prior to embryo transfer. Differences in implantation, clinical pregnancy, and miscarriage rates of cycles with and without PRP infusions were analyzed.

**Results:**

The implantation rate seen in the PRP group was 6.7%. No significant difference was found the between the implantation, clinical pregnancy, ongoing pregnancy, and miscarriage rates of frozen-thawed embryo transfers with and without PRP infusion. However, the effect size of PRP infusion (Cohen's d=0.39) on implantation rates revealed a relationship in medium strength.

**Conclusion:**

Platelet-rich plasma might potentially yield beneficial effects as a safe therapeutic option offered alongside other treatments designed to improve the reproductive outcomes of women with repeated implantation failure.

## INTRODUCTION

According to Coughlan *et al*. (2014), recurrent implantation failure (RIF) is defined as failure to achieve clinical pregnancy after a minimum of three fresh or frozen transfer cycles in women under the age of 40 years verified within five weeks or more on transvaginal sonography (TVS). Lack of proper synchronization of the developing embryo with the endometrium plays an important role, regardless of other relevant causes of embryo implantation failure such as poor embryo quality, uterine anomaly, infection, immunological or thromboembolic factors and insufficient hormone levels (Coughlan *et al.,* 2014; Richter *et al*., 2007; Simon & Laufer, 2012; Ly *et al*., 2010; Timeva *et al*., 2014). The techniques currently used to treat patients with RIF include blastocyst transfer, assisted hatching, salpingectomy, hysteroscopy, endometrial scratch, gamete donation, surrogacy, thrombophilia and immunologic treatment, and growing endometrial thickness (Coughlan *et al*., 2014; Simon & Laufer, 2012). Nevertheless, a significant portion of patients with poor-quality endometria does not respond to treatment.

Platelet-rich plasma (PRP) is a relatively safe and noninvasive therapy recently used in women with a history of RIF. It seems that the main use of PRP for RIF resides in improving the thickness and vascularity of suboptimal endometria to facilitate embryo implantation (Tandulwadkar *et al*., 2017; Zadehmodarres *et al*., 2017). Its therapeutic effect derives from the natural activation of cytokines and the presence of growth factors (GFs). PDFG and TGF-β are known for their vital roles in healing damaged tissues. Preparations with concentrations greater than 1,000,000 platelets per microliter were made from patient blood samples. The risk of allergic reactions or infection by hepatitis virus or HIV was eliminated since synthetic materials were not used in the preparations (Bos-Mikich *et al*., 2018; Alves & Grimalt, 2018; Martínez-Zamora *et al*., 2011).

In spite of the widespread use of PRP in various fields in medicine and its proven safety, very few studies have addressed the efficacy of PRP in the treatment of RIF. In a seminal trial, Chang *et al*. (2015) conducted a pilot study and showed that PRP improved the pregnancy outcomes of IVF patients. Other studies subsequently reported conflicting viewpoints on the efficacy of PRP at improving the endometrial thickness and implantation outcomes of women undergoing IVF. A handful of studies have looked into the role of PRP in the reproductive outcome of women with a history of RIF (Timeva *et al*., 2014; Chang *et al*., 2015; Cai *et al*., 2011; Faramini *et al*., 2017). The definitions of RIF and the exclusion criteria adopted in these studies were often conflicting (Vlachadis *et al*., 2014).

ART centers struggle with cases of RIF and unsuccessful IVF imposes a significant psychological and financial burden on infertile couples. This study aimed to assess the role of platelet-rich plasma (PRP) in the reproductive outcomes of patients with repeated implantation failure (RIF) undergoing ICSI cycles.

## MATERIALS AND METHODS

### Participants

The Research Ethics Committee of the Babol University of Medical Sciences approved this study. This historically controlled study was registered with the Iran Registry of Clinical Trials (IRCT) and assigned number 20180417039338N2.

Thirty females were selected from frozen-thawed embryo transfer candidates seen from June 2017 to August 2018 at the Fatemezahra Infertility Research Center affiliated with the Babol University of Medical Sciences. Recurrent implantation failure (RIF) was defined as the non-visualization on TVS of a gestational sac within five weeks or more of ICSI after three embryo transfers (Coughlan *et al*., 2014). The study included only patients submitted to ICSI more than four times in order to minimize potential bias toward the occurrence of RIF. Only failed attempts to get pregnant with the same partner were reported. Unfortunately, for ethical considerations the study had a pre-post design. Since the previous papers described pilot studies, we included all women with RIF in our center who were candidates for frozen-thawed embryo transfers. It is worth mentioning that PRP is not routinely employed in our center in individuals with an endometrial thickness of less than 7 mm. All embryo recipients with inadequate endometrial development are given oral estradiol valerate tablets.

The study included healthy women aged 18-40 years with a BMI <30 Kg/m^2^ and a history of four or more episodes of implantation failure submitted to frozen-thawed embryo transfers possessing excellent or good embryos.

Women with hemoglobin disorders (hemoglobin lower than 11 mg/ml and platelet count below 150,000), immune or endocrine disorders, genetic or chromosomal abnormalities, uterine infection or anomaly, history of uterine incisions including myomectomies and cesarean sections, fever, malignancy, females on NSAIDs 10 days prior to the intervention or on corticosteroids over the past month, smokers, and women with the medium or poor-quality frozen-thawed embryos were excluded. The thirty patients deemed eligible gave written consent to joining the study. The cycle each patient underwent immediately before the administration of PRP was considered a control cycle.

### Processing

Demographic and medical data were collected from the patients' records. One month before the start of the study, all included patients underwent hysteroscopy to verify whether they had uterine anomalies.

All participants were prescribed the conventional long protocol in their previous cycles and the retrieved oocytes were inseminated with sperm from their partners. Frozen embryos of higher quality were stored and transferred during the course of the study. As part of endometrial preparation, patients with RIF were given 6 mg of estradiol/day (Abu Rayhan, 2 mg tablet, Iran) for eight days starting on the second or third day of the menstrual cycle. TVS was performed on day 9-10 of the cycle. Patients with an endometrial thickness of 7 mm had PRP administered to them on the same day, and the laboratory was told to initiate embryo warming.

Forty-eight to 72 hours following warming, two good quality embryos were transferred. Patients were also given two progesterone suppositories per day (Cycloghist, 400 mg, Actavis, UK) from the day of PRP to the day of embryo transfer (approximately for 2-3 days, depending on embryo thawing and growth). If endometrial thickness was below 7 mm in TVS, the dosage of estradiol was increased to 8mg for two days and TVS was repeated. The dosage of estradiol was increased by 2mg every other day, and PRP was infused when the endometrium reached a thickness of 7mm. If endometrial thickness failed to increase after a first infusion of PRP, a second infusion was performed 48 hours later. If endometrium thickness remained below 7mm until day 20 of treatment, the cycle was canceled but the patient was not removed from the study. Estradiol and progesterone were kept at the same respective doses from the day of embryo transfer until pregnancy was observed. Patients with positive B-HCG tests were kept on the protocol until the 12^th^ week of gestation unless they had a miscarriage.

### PRP preparation

Pure platelet-rich plasma (P-PRP) without leukocytes and low-density fibrin were used in the preparation (Dhurat & Sukesh, 2014). A volume of 15-20 cc of the patient's own blood was drawn and placed into blood collection tubes with 2.5 cc of an anticoagulant agent (Merck) under sterile conditions. To avoid platelet activation during blood centrifugation, the temperature must be kept at 21˚C-24˚C. The blood samples were centrifuged (Becton Dickinson, UK) at 1200 rpm for 12 minutes. After blood separated into three layers - platelets on top, white blood cells in the middle, and red blood cells in the bottom layer - the upper and middle layers were transferred to another sterile tube and were centrifuged again at 3300 rpm for seven minutes, with erythrocytes and platelets settling on the bottom of the tube. The upper two thirds of plasma containing platelet-poor plasma were discarded. The lower third of plasma (0.5-0.7mL) was homogenized and used to produce a platelet-rich plasma preparation with approximately five times more platelets than whole blood. The PRP preparation was infused into the uterine cavity of the patients with the aid of an intrauterine insemination catheter (Takvin co, Iran).

### Assessment of outcomes

Primary outcome implantation rate was defined as the number of gestational sacs seen in TVS two to three weeks following embryo transfer divided by the number of embryos transferred (Sullivan *et al.*, 2013). Clinical pregnancy rate was measured as a secondary outcome. Clinical pregnancy was defined as viable intrauterine pregnancy seen in TVS after 5-6 weeks of embryo transfer (Zegers-Hochschild *et al*., 2009). Miscarriage rate was also measured as a secondary outcome. Miscarriage was defined as pregnancy terminated before 20 weeks of gestation.

### Statistical analysis

An intention-to-treat strategy was adopted in statistical analysis to find implantation and clinical pregnancy rates per cycle. The Statistical Package for Social Sciences (SPSS) version 18.0 was used. McNemar's test was used to verify the existence of differences on a dependent variable in the group before and after the intervention. Values were considered statistically significant when *p*<0.05. The effect size of treatment (Cohen's d) was calculated according to Durlak (2009). The BESD (binomial effect-size display) measurement was done as described by Rosenthal & Rubin (1982). The number of patients that needed to be treated for one of them to benefit from the intervention with PRP was calculated according to Furukawa & Leucht (2011).

## RESULTS

The study included 36 women with RIF. Three individuals were excluded for not having high quality frozen-thawed embryos, one for having a history of uterine incision, and one for having severe thyroid disorder. One woman refused to join the study. Thirty women were ultimately included. One woman had her cycle cancelled for lack of endometrial growth until day 20 of the cycle, but she was not excluded from the study.

Table 1 shows demographic data and preliminary criteria. An implantation rate of 6.7% was seen in the PRP group, as shown in Table 2. In previous cycles, the patients with RIF were unable to get pregnant. The results derived from the analysis indicated the absence of a significant difference between the implantation, clinical pregnancy, ongoing pregnancy, and miscarriage rates of frozen-thawed embryo recipients treated with and without PRP (*p*>0.05) (Table 2). However, the difference in the implantation rate before and after treatment with PRP yielded an effect size of 0.39 (Cohen's d). An increase of 6.7% was seen in intervention success among women receiving frozen-thawed embryos using PRP when compared to individuals not given RRP (0%) (Binomial Effect Size Display, BESD). The number of patients needed to treat (NNT) to attain one successful implantation was 15.

**Table 1:** Baseline characteristics of women with RIF administered PRP

N		30
Age (years)		33.3±4.36
BMI		26.7±2.96
Cause of Infertility (n)	Female	5 (16.7)
	Male	6 (20)
	Both	19 (63.3)
Type of infertility (n)	Primary	5 (16.7)
	Secondary	25 (83.3)
Infertility duration (years)		11.3±1.58
Previous failed ICSI cycle (n)		4.2±0.73
Endometrial thickness 48h after PRP(mm)		7.79±1.05
Days of endometrial preparation		9.66±1.68
Number of PRP	One	26 (86.7)
	Two	4 (13.3)
Transferred Embryo (n)		2.6±0.23

The values in the table were presented as Mean±SD and n(%).

**Table 2:** Reproductive outcome of women with RIF administered PRP

Criteria	With PRP n (%)	*P*-value
Positive chemical pregnancy	4 (13.3)	0.12
Implantation Rate	2 (6.7)	0.16
Clinical pregnancy	1 (3.3)	1
Ongoing pregnancy	1 (3.3)	1

McNemar's test was used for comparison.

The mean difference is significant if *p*<0.05.

The clinical pregnancy rate in the PRP group was 3.33% (Table 2). The effect size of treatment to produce a difference on the clinical pregnancy rates of frozen-thawed embryo recipients before and after PRP was 0.27 (Cohen's d). PRP also increased the clinical pregnancy success to 3.4% (BESD) in the recipients of the frozen-thawed embryo. Thirty patients needed to be treated (NNT) with PRP to achieve one clinical pregnancy.

## DISCUSSION

Although our findings indicated the lack of a significant difference in the implantation rates of frozen-thawed embryo recipients treated with and without PRP, the measured effect size of treatment revealed a relationship of medium strength (Cohen's d categories; <.1: no effect; .1 to .3: small effect; .3 to .5: intermediate effect; >.5: large effect). In addition, the binomial effect size display (BESD) indicated a potential practical importance of the effect size of 6.7. According to these data, we might infer that PRP treatment produced at least 6.7% implantation success in women with RIF receiving frozen-thawed embryos when compared to women undergoing cycles without PRP (implantation rate = 0%). The non-significant p-value calculated is probably linked to the lack of an adequate sample size, a clear limitation of our study. Although our study was based on a small sample of individuals, the observed implantation rate was confirmed by the magnitude of the difference rather than confounded by the small sample size.

In a way, this result reflects the findings reported by Farimani *et al*. (2016) and Tandulwadkar *et al*. (2017), although overall levels were lower than previously reported levels (clinical pregnancy rate: 66.6% and 45.31%, respectively). Tandulwadkar *et al*. (2017) investigated patients with suboptimal endometrial parameters, not individuals with RIF. Farimani *et al*. (2016) studied patients with RIF, but the method adopted in their study and the protocol used to produce the PRP preparation were not fully described. Eftekhar *et al*. (2018) investigated poor endometrial response in a study with women given standard hormone replacement therapy and reported an implantation rate of 21%. The definition of RIF used in our study was different from the definition adopted by Eftekhar *et al*. (2018). These authors included women undergoing frozen embryo transfers for poor endometrial response. It is important to consider the possible methodological differences between the interventional studies cited above and the present study.

The decrease in the implantation rate observed in the present study might be attributed to limitations in the adopted exclusion criteria. Patients with thrombotic defects were not excluded, showing an evident weakness of the study. According to previous studies, thrombophilia might hamper the implantation of the embryo in women experiencing repeated ART failures (Di Nisio *et al*., 2011; Qublan *et al*., 2006).

Another uncontrolled factor present in our study was its pre-post nature, a potential source of bias. The study cannot clearly answer whether pregnancy outcomes might improve or deteriorate with the treatment. The results reported in this study must be interpreted with caution.

A variety of factors beyond the egg, embryo, and endometrium contribute to repeated implantation failure (Penzias, 2012). Further randomized controlled trials with sound inclusion and exclusion criteria are required to analyze the variables involved and shed light on the efficacy of PRP at improving reproductive outcomes.

## CONCLUSION

Despite its limitations and considering the strength of the effect of PRP in patients with RIF patients, this study offered valuable insight into the use of platelet-rich plasma as an option to improve embryo implantation in patients with a history of RIF. Future studies are required to provide more definitive evidence.
